# Barriers and opportunities: Intercellular mitochondrial transfer for cardiac protection—Delivery by extracellular vesicles

**DOI:** 10.3389/fcvm.2022.1024481

**Published:** 2023-01-04

**Authors:** Tian Chen, Naifeng Liu

**Affiliations:** Department of Cardiology, School of Medicine, Zhongda Hospital, Southeast University, Nanjing, China

**Keywords:** mitochondria delivery, mitochondrial quality control, extracellular vesicles, cardioprotection, intercellular crosstalk

## Introduction

Mitochondria are the powerhouse of cardiomyocytes to maintain a sufficient energy supply for the strenuous mechanical workload. Mitochondrial dysfunction and consequent reduced ATP generation have thus a significant deleterious impact on heart function. In addition to their role as the cell's powerhouse, mitochondria are also central to several other activities, including calcium storage, production of reactive oxygen species, regulation of metabolic responses, and cell death. Irreversible mitochondrial defects make the relevant heart diseases only amenable to palliative treatments. As a result, the cell has evolved mechanisms to enable the clearance of compromised mitochondria, such as mitophagy, and repaired by the expulsion of mitochondria-derived vesicles (MDVs) containing damaged cellular components (1). In this article, we emphasize the barriers and opportunities of a new mitochondrial quality control process that enables the clearance of compromised mitochondria through extracellular vesicles to increase cell fitness at the heart.

## Mitochondrial transfer through different cell-derived EVs

A landmark study details a novel way by which cardiomyocytes eject “cardiac exophers”, potentially new types of extracellular vesicles (EVs) containing damaged dysfunctional mitochondria into the interstitium, where they are actively phagocytosed by neighboring cardiac resident macrophages (cMacs) through the surface phosphatidylserine (PS) signal, coupling the cell-autonomous autophagy to phagocyte-mediated heterophagy (2). This process of mitochondrial disposal through these subcellular particles occurs in a range of pathophysiological conditions and is enhanced by intense stress to maintain both myocyte and interstitial homeostasis and avoid inflammasome activation. It is noteworthy that these vesiculated fragments do not stain with nuclear DNA indicator DAPI. In addition, properties including size, cargo, and mechanism of formation distinguish them from classical extracellular vesicles. Most notable is their ability to mediate intercellular transport of cytosolic cargo that is more likely to contain dysfunctional and potentially harmful organelles.

The transfer of compromised mitochondria from cardiomyocytes to cMacs appears to be tissue-specific. Other long-lived, post-mitotic cells without the ability of common self-renewal and regeneration pathways, such as neurons and skeletal myocytes, which have high mitochondrial content and remarkable metabolic activity, rely heavily on waste disposal systems for quality control. Evidence is accumulating that intercellular mitochondria transfer to macrophages mediates systemic metabolic homeostasis. For example, a recent comprehensive study demonstrates that adipose tissue-resident macrophages acquire mitochondria from neighboring adipocytes (3). Genetic disruption of this process exhibits lower energy expenditure and more diet-induced obesity (3). In the case of adipocytes, Crewe et al. recently demonstrated that EVs that evade tissue macrophages can enter circulation and be taken up by cardiomyocytes, a process that plays a key role in cardiac oxidative stress through the physical transfer of damaged mitochondria *via* EVs and has an unexpected protective effect against ischemia/reperfusion injury (4). These findings highlight the proximal or distal effects of different cell-derived EVs and mitochondrial dysfunction in inter-tissue or inter-organ crosstalk and the onset of metabolic deterioration.

Mitocytosis, a migrasome-dependent mechanism for releasing cellular contents and allowing defective mitochondria to migrate to the trailing edge of the plasma membrane, has recently been found to involve in regulating the maintenance of mitochondrial homeostasis in cells (5). The authors named these mitochondrion-containing migrasomes “mitosomes”. Of note, migrasomes are originated from immune cells, metastatic tumor cells, and other migrating cells. Although the role of mitosomes in cardiovascular physiology and pathology is unclear, the migration of immune cells occurs during immune responses and cell development, which could readily speculate that mitosomes may exert relevant beneficial effects in many biological processes such as angiogenesis, wound healing, and tissue regeneration to ensure the maintenance of optimal mitochondrial performance. Table 1 summarizes studies reported so far on an impaired mitochondrial transfer through EV's transfer to other cell types.

**Table 1:** Summary of damaged mitochondrial transfer-related EVs.

**EVs**	**Donor cells**	**Recipient cells**	**Compounds**	**Effects**	**Clearance pathways**	**References**
Microvesicles	Bone marrow-derived stromal cells	Epithelia	Dysfunctional mitochondria	Protect against acute lung injury	Engulfed by the epithelium	(6)
Microvesicles	Mesenchymal stem cells	Macrophages	Partially depolarized mitochondria	Manage intracellular oxidative stress	Fused by macrophages	(7)
Exosomes	Bronchoalveolar lavage myeloid-derived regulatory cells	T cells	Polarized mitochondria	Generate reactive oxygen species (ROS), change pro-inflammatory function and signaling	Not mentioned	(8)
Exophers	Cardiomyocytes	Cardiac-resident macrophages	Defective mitochondrial particles	Support heart function and metabolism	Captured and eliminated by cardiac-resident macrophages	(2)
Small extracellular vesicles	Adipocytes	Cardiomyocytes	Damaged mitochondrial particles	Increase ROS in cardiomyocytes	Taken up by cardiomyocytes	(4)
Migrasomes	Migrating cells	Position at the cell periphery	Damaged mitochondria particles	Preservation of metabolic stability	Released into the extracellular space or directly taken up by surrounding cells	(5)

## Therapeutic significance of mitochondrial transfer

The ability of these endogenous vesicles to survive in the extracellular space, bypass biological barriers, and deliver their bioactive molecules to recipient cells gives them great therapeutic potential. EVs with cardiovascular efficacy have been isolated from various cell sources, such as mesenchymal stem cells, putative cardiac progenitors, pluripotent stem cell-differentiated cells, and differentiated somatic cells (9). The therapeutic effects of EVs are mainly attributed to the delivery of proteins and/or noncoding RNAs, especially miRNAs. Although the mechanism of mitochondrial protein and mtDNA loading in different EVs remains unknown, the recognition of intercellular mitochondria transfers through EVs has opened up promising therapeutic perspectives. Ikeda et al. present a novel strategy in which human-induced pluripotent stem cell-derived cardiomyocytes (iCMs) derived EVs can effectively transfer mitochondria from progenitor cells to cardiomyocytes *in vitro*, and this process has been related to therapeutic effects in animal models of ischemia/ reperfusion injury (10). Isolated mitochondria showed no beneficial effects on cardiomyocytes, highlighting the key role of EVs to release their cargo and exerting relevant beneficial effects by facilitating the quick transfer of mitochondrial cargo into the recipient iCMs and conferring marked resistance to extracellular damage.

Boudreau et al. found that a pro-inflammatory setting (LPS stimulation) led to the release of free and encapsulated active mitochondria (11), and studies reporting the selective release of mitochondrial content mainly used resting non-immune cell lines, suggesting that cellular conditions may greatly affect the release of mitochondrial EV content. It has long been known that cells, especially mesenchymal stem cells, are capable of transferring mitochondrial content to cells lacking mtDNA, thereby rescuing their metabolic activity. However, very few studies have taken advantage of the cellular machinery to engineer EVs with specific epitopes to target the heart. More research on different EV sources and how the cellular environment affects mitochondrial release could help regulate the nature and quantity of EVs containing mitochondria and develop new therapies for diseases related to mitochondrial dysfunction.

Autogenous utilization of EVs is feasible under certain conditions. However, most applications are likely to use well-established non-autologous EVs primarily due to demand, safety concerns, and commercial requirements. It is with these considerations that creative and audacious regenerative ways have been proposed in pursuit of designing EV-based therapeutic delivery systems, such as using genetically engineered cell and EV-inspired liposomes, or post-modifying with drugs or surface ligands (12). Non-engineered and non-autologous EVs have been used in human subjects in many clinical studies with promising safety results (13). The limitations of EVs in cardiovascular therapeutics relate to the lack of tools to effectively target the injured myocardium. Several professional recommendations have been raised to assist the clinical translation of EVs, which involve minimal experimental guidelines, transparency tools, reference materials, validation, etc. (14). However, massive efforts are required for fulfilling the clinical application, whether it is to mature endogenous EVs to transport damaged mitochondria or to prepare exogenous EVs containing healthy mitochondria.

## Barriers and opportunities

As mentioned previously, the existence of parenchymal-immune crosstalk further highlights the critical role of macrophages in maintaining cardiac homeostasis (15). Depending on their strong phagocytic capacity, macrophages closely cooperate with tissue-resident cells to eliminate broken mitochondria. Interestingly, previous data suggest differences in phagocytosis efficiency between cMac subsets (16). This may be interrogated by using comprehensive lineage tracing and fate mapping studies. Functional mitochondria might be required to supply energy for EV transferring or degradation. Although it is unclear how garbage is distinguished and classified, cells may balance the energy demand for cellular repair and that required for mitochondrial release. Whether a specific threshold of garbage accumulation is required to be reached to generate EVs remains poorly understood, although intense mechanical stress and functionally compromised cardiomyocytes can increase the trash extrusion response. Moreover, how macrophages can sense the presence of EVs and secondarily impact overall mitochondrial function or tissue maintenance in homeostatic vs. injury environments remains to be explored further. Nevertheless, the remarkably plastic nature and multifaceted origins of macrophages indicate that more unexpected physiological functions will emerge in the future (17).

The nomenclature and definition of EVs have been a hot topic in the field due to their different biogenesis, morphology, composition, and secretion. Transmission electron microscopy (TEM) has been the preferred technique to directly observe the size and morphology of EVs, but the visualized analysis still remains a major challenge because of the uncertain identification of the optimal parameters and subjective defining. EVs offer great clinical potential, with applications in both diagnostics and therapeutics. Technologies have been developed to track EVs *in vivo* and follow their biodistribution [e.g., luminescence imaging techniques and methods that rely on PET-MRI (18) or SPECT (19)]. Moreover, bioengineering of EVs can enrich their therapeutic contents, control their spatial and temporal release, and improve their internalization and capacity for on-target binding (9).

The possibility of designing EV-based therapeutic delivery systems to freely clear damaged mitochondria or transplant healthy mitochondria into failing hearts will represent a revolution in regenerating and repairing hearts with aberrant metabolism. Several issues should be solved related to the translational potential of this approach: (I) the spatial and temporal information of released EVs and their coupling with the mitophagy pathway, (II) the viability of transferred mitochondria in interstitial space or into recipient cells, (III) the mechanism of degradation or internalization of defective mitochondria, and (IV) the adjustable approach of communication between cardiac working cells and EVs. Moreover, given the harsh environment of the injured myocardium (i.e., inflammatory, hypoxic, and proapoptotic conditions), poor transplantation results may hinder its clinical applications.

## Conclusion

The intercellular transport of mitochondria attempts to eliminate the preconceptions of mitochondria and mtDNA segregation and inheritance. This mitochondrial plasticity may play a great role in cardiac tissue homeostasis, development, and aging. The development of mitochondrial delivery protocols is a critical task in translating recent discoveries into appropriate clinical applications.

## Author contributions

TC wrote and was responsible for the manuscript content. NL was responsible for reviewing the manuscript and making final decisions about this article. All authors contributed to the article and approved the submitted version.

## References

[1] Green DR. Mitochondrial quality control: just walk away. Cell Metab. (2021) 33:1069–71. 10.1016/j.cmet.2021.05.011 34077713

[2] Nicolas-Avila JA Lechuga-Vieco AV Esteban-Martinez L Sanchez-Diaz M Diaz-Garcia E Santiago DJ. A network of macrophages supports mitochondrial homeostasis in the heart. Cell. (2020) 183:94–109. 10.1016/j.cell.2020.08.031 32937105

[3] Brestoff JR Wilen CB Moley JR Li Y Zou W Malvin NP. Intercellular mitochondria transfer to macrophages regulates white adipose tissue homeostasis and is impaired in obesity. Cell Metab. (2021) 33:270–82. 10.1016/j.cmet.2020.11.008 33278339 PMC7858234

[4] Crewe C Funcke JB Li S Joffin N Gliniak CM Ghaben AL. Extracellular vesicle-based interorgan transport of mitochondria from energetically stressed adipocytes. Cell Metab. (2021) 33:1853–68. 10.1016/j.cmet.2021.08.002 34418352 PMC8429176

[5] Jiao H Jiang D Hu X Du W Ji L Yang Y. Mitocytosis, a migrasome-mediated mitochondrial quality-control process. Cell. (2021) 184:2896–910. 10.1016/j.cell.2021.04.027 34048705

[6] Islam MN Das SR Emin MT Wei M Sun L Westphalen K. Mitochondrial transfer from bone-marrow-derived stromal cells to pulmonary alveoli protects against acute lung injury. Nat Med. (2012) 18:759–65. 10.1038/nm.2736 22504485 PMC3727429

[7] Phinney DG Di Giuseppe M Njah J Sala E Shiva S St Croix CM. Mesenchymal stem cells use extracellular vesicles to outsource mitophagy and shuttle microRNAs. Nat Commun. (2015) 6:8472. 10.1038/ncomms9472 26442449 PMC4598952

[8] Hough KP Trevor JL Strenkowski JG Wang Y Chacko BK Tousif S. Exosomal transfer of mitochondria from airway myeloid-derived regulatory cells to T cells. Redox Biol. (2018) 18:54–64. 10.1016/j.redox.2018.06.009 29986209 PMC6031096

[9] de Abreu RC Fernandes H da Costa Martins PA Sahoo S Emanueli C Ferreira L. Native and bioengineered extracellular vesicles for cardiovascular therapeutics. Nat Rev Cardiol. (2020) 17:685–97. 10.1038/s41569-020-0389-5 32483304 PMC7874903

[10] Ikeda G Santoso MR Tada Y Li AM Vaskova E Jung JH. Mitochondria-rich extracellular vesicles from autologous stem cell-derived cardiomyocytes restore energetics of ischemic myocardium. J Am Coll Cardiol. (2021) 77:1073–88. 10.1016/j.jacc.2020.12.060 33632482 PMC8626617

[11] Boudreau LH Duchez AC Cloutier N Soulet D Martin N Bollinger J. Platelets release mitochondria serving as substrate for bactericidal group IIA-secreted phospholipase A2 to promote inflammation. Blood. (2014) 124:2173–83. 10.1182/blood-2014-05-573543 25082876 PMC4260364

[12] Herrmann IK Wood MJA Fuhrmann G. Extracellular vesicles as a next-generation drug delivery platform. Nat Nanotechnol. (2021) 16:748–59. 10.1038/s41565-021-00931-2 34211166

[13] Lener T Gimona M Aigner L Börger V Buzas E Camussi G. Applying extracellular vesicles based therapeutics in clinical trials - an ISEV position paper. J Extracell Vesicles. (2015) 4:30087. 10.3402/jev.v4.30087 26725829 PMC4698466

[14] De Wever O Hendrix A. A supporting ecosystem to mature extracellular vesicles into clinical application. EMBO J. (2019) 38:e101412. 10.15252/embj.2018101412 30975688 PMC6484403

[15] Swirski FK Nahrendorf M. Cardioimmunology: the immune system in cardiac homeostasis and disease. Nat Rev Immunol. (2018) 18:733–44. 10.1038/s41577-018-0065-8 30228378

[16] Epelman S Lavine KJ Beaudin AE Sojka DK Carrero JA Calderon B. Embryonic and adult-derived resident cardiac macrophages are maintained through distinct mechanisms at steady state and during inflammation. Immunity. (2014) 40:91–104. 10.1016/j.immuni.2013.11.019 24439267 PMC3923301

[17] Wynn TA Chawla A Pollard JW. Macrophage biology in development, homeostasis and disease. Nature. (2013) 496:445–55. 10.1038/nature12034 23619691 PMC3725458

[18] Banerjee A Alves V Rondão T Sereno J Neves Â Lino M. A positron-emission tomography (PET)/magnetic resonance imaging (MRI) platform to track in vivo small extracellular vesicles. Nanoscale. (2019) 11:13243–8. 10.1039/c9nr02512j 31290510

[19] Hwang DW Choi H Jang SC Yoo MY Park JY Choi NE. Noninvasive imaging of radiolabeled exosome-mimetic nanovesicle using (99m) Tc-HMPAO. Sci Rep. (2015) 5:15636. 10.1038/srep15636 26497063 PMC4620485

